# No evidence on infectious DNA-based agents in pediatric acute lymphoblastic leukemia using whole metagenome shotgun sequencing

**DOI:** 10.3389/fcimb.2024.1355787

**Published:** 2024-06-21

**Authors:** Amadeus T. Heinz, Silke Grumaz, Christoph Slavetinsky, Michaela Döring, Manon Queudeville, Rupert Handgretinger, Martin Ebinger

**Affiliations:** ^1^ Department of Pediatric Hematology and Oncology, University Children´s Hospital Tuebingen, Tuebingen, Germany; ^2^ Stuttgart Cancer Center, Zentrum für Kinder-, Jugend- und Frauenmedizin (Olgahospital), Pädiatrie 5 (Pädiatrische Onkologie, Hämatologie, Immunologie), Klinikum der Landeshauptstadt Stuttgart, Stuttgart, Germany; ^3^ Noscendo GmbH, Duisburg, Germany; ^4^ Department of Pediatric Surgery and Urology, University Children´s Hospital Tuebingen, Tuebingen, Germany; ^5^ Department for Stem Cell Transplantation and Immunology, Klinikum Hamburg-Eppendorf (UKE), Hamburg, Germany; ^6^ Abu Dhabi Stem Cells Center, Abu Dhabi, United Arab Emirates

**Keywords:** high-throughput sequencing, ALL, shotgun sequencing, infectious etiology, microbiome

## Abstract

The etiology of pediatric acute lymphatic leukemia (ALL) is still unclear. Whole-metagenome shotgun sequencing of bone marrow samples in patients with treatment-naïve ALL (n=6) was performed for untargeted investigation of bacterial and viral DNA. The control group consisted of healthy children (n=4) and children with non-oncologic diseases (n=2) undergoing bone marrow sampling. Peripheral blood of all participants was investigated at the same time. After bioinformatical elimination of potential contaminants by comparison with the employed controls, no significant amounts of microbial or viral DNA were identified.

## 1 Introduction

Acute lymphoblastic leukemia (ALL) is the most common childhood cancer (Pui, 1995). Nevertheless, its etiology is still unknown. Derived from epidemiological studies, infection-related hypotheses have frequently been discussed in the past (Greaves and Alexander, 1993; Kinlen et al., 1995). However, no specific causative agent has been identified, so far (O’Connor and Boneva, 2007). Using next-generation sequencing (NGS) techniques for untargeted amplification of the whole viral and bacterial metagenome in pre-treatment bone marrow samples, a higher viral load has been identified in pediatric patients with ALL compared to acute myeloid leukemia (AML) (Francis et al., 2014). To further investigate the infection-related hypothesis in pediatric ALL using novel sequencing techniques, we performed a pilot trial to search for microbial and viral DNA using whole metagenome sequencing (WMS) of peripheral blood and bone marrow samples of six patients with treatment-naïve ALL and six children without oncologic diseases acting as negative controls.

## 2 Methods

### 2.1 Collection of samples

All samples were collected during planned bone marrow punctures of the iliac crest after disinfection of the skin and under sterile conditions using an aspiration cannula. Bone marrow punctures were scheduled in six patients for initial diagnosis of ALL, for bone marrow donation in four healthy participants and for other reasons in two participants (diagnostic purpose in one patient with severe aplastic anemia (SAA) and gathering of an autologous backup ahead of bone marrow transplantation in one patient suffering from sickle cell anemia). All work was conducted within the formal approval of the institutional ethics comittee (University Tuebingen, approval number 561/2020BO1, gathered 11/2020) and in accordance with the Declaration of Helsinki. Blood and bone marrow samples were collected after obtaining written informed consent of participant’s guardians and informing study participants according to the corresponding age group.

The sample collection for this study was performed in the midst or the end of the bone marrow aspiration, not at the beginning, to minimize the risk of contamination through commensal bacteria of the skin. A total of five milliliters of bone marrow was drawn, plus one additional milliliter of bone marrow for microbial cultivation as contamination control. Five milliliters of peripheral blood were collected through a peripheral venous blood draw or a central venous catheter (Hickman line). In patients below three years of age, two milliliters of blood were taken. Collection of bone marrow and peripheral blood was performed simultaneously, whenever possible.

The collected sample material was placed in DNA stabilizer tubes (Streck Cell-free DNA BCT, CE-IVD, Streck, La Vista, NE, USA) and sent to the laboratory at room temperature. After centrifugation for separation of plasma and peripheral blood cells, the plasma was stored at -80°C until further processing. Bone marrow samples were treated in the same manner.

### 2.2 Metagenomic next-generation sequencing

Blood samples and bone marrow samples were processed in a blinded manner and therefore processed identically. Cells were separated from the supernatant by centrifugation at 1,600 x g for 10 min at 4°C and the supernatant was transferred to a fresh reaction tube. Then, a second centrifugation step at 16,000 x g for 10 min at 4°C was performed, supernatants were again transferred, and aliquots were further stored at -80°C. Nucleic acid isolation, quality controls and library preparation were carried out as previously described (Grumaz et al., 2016). NGS was performed on a Illumina ^©^ NextSeq 550 instrument. Adequate positive and negative controls accompanied all laboratory and sequencing procedures. Raw sequencing data were subjected to various QC controls comprising PHRED-Score filtering, adapter trimming, complexity filtering as well as k-mer based classification and contamination screening. To pass the quality filter, read quality needed to surpass a PHRED score of 20 and achieve a minimal length of 50 bp after quality control. All data generated were analyzed using Noscendo’s DISQVER platform, integrating the CE-IVD for pathogen detection assay from blood. The DISQVER platform comprises a curated microbial genome reference database of over 16,000 microbial species covering >1,500 pathogens and can detect bacteria, DNA-viruses, fungi, and parasites while differentiating contaminating commensals from infective agents via run controls and dedicated control collectives. For each pathogen type (bacterium, virus, fungus, parasite) a dedicated minimum threshold for reporting was set, that is further refined by incorporating data for each species individually regarding their observation in control cohorts. The limit of detection (LOD) was determined by bioinformatic and wetlab analytical validation procedures, which are further described in **Supplementary Material 1**. For the PCA analysis, raw sequencing data after removal of human sequences and after classification of the non-human fraction was used for each sample. Read counts were log10 transformed prior to PCA analysis. The positive control was used after bioinformatic exclusion of the known, spiked-in microbial sequences, in order to not bias the results by taking these into account.

### 2.3 Statistical methods

Statistical analyses were conducted using SPSS 27 (Armonk, New York) and RStudio 2022.12.0 (Boston, MA). Descriptive statistics are reported as median [range], if not otherwise specified. For comparison of means between cfDNA levels in control and ALL cohorts, the Wilcoxon rank sum test was used as values were not-normally distributed. For comparison of read mapping proportions between the different groups, the Kruskal-Wallis test was used.

## 3 Results

### 3.1 Participant characteristics

As shown in **Table 1**, participants were well balanced concerning gender and age. Common type ALL was the most frequent diagnosis in the ALL group, whereas controls were mainly healthy children and young adolescents willing to donate bone marrow for a sibling.

**Table 1:** Participant characteristics.

	ALL (n=6)	Control group (n=6)
Diagnosis	- cALL (n=4) - Ph+ ALL (n=1) - Pro-B-ALL (n=1)	- Healthy donor for bone marrow transplantation (n=4) - Sickle cell anemia (n=1) - Severe aplastic anemia (n=1)
Sex (male/female)	4/2	3/3
Age (mean [range])	9.4 years [3.6 – 16.8]	10.3 years [2.4 – 17.3]
Antibiotics 8 weeks ahead of BMA [% of participants]	4/6 [67]	2/6 [33]
Type of antibiotic treatment	Piperacillin/Tazobactam (n=2) Cefuroxim (n=2)	Piperacillin/Tazobactam (n=1) Penicillin (n=1)
Mean cfDNA isolated in BM [ng/µl; range]	4.78 [1.4 – 11.8]	2.03 [0.9 – 3.1]
Mean cfDNA isolated in PB [ng/µl; range]	2.12 [0.09 – 8.8]	0.33 [0.07 – 1.5]

BMA: bone marrow aspiration; cALL: common-type ALL, cfDNA: cell free DNA; Ph+ ALL: Philadelphia-positive (BCR-ABL) ALL.

In the ALL group, four out of six patients received antibiotic treatment ahead of BMA. In three patients, intravenous Piperacillin/Tazobactam or Cefuroxime plus Clindamycin was administered just one day ahead of BMA. The remaining case with fever and mild pancytopenia was treated intravenously for six days with Cefuroxime and then discharged. Fourteen days later, ALL was diagnosed via BMA.

In the control group, only the two patients suffering from SAA or sickle cell anemia received antibiotics ahead of BMA: Piperacillin/Tazobactam in the SAA patient two days ahead of BMA, and long-term Penicillin prophylaxis in the sickle cell patient.

### 3.2 Results of WMS

In total, an mean of 4.8 ng/µl of cell free DNA (cfDNA) could be isolated in the bone marrow of the ALL patients, compared to 2 ng/µl in the control group (**Table 1**, p=0.18). In peripheral blood, mean cfDNA concentration was 2.1 ng/µl in ALL and 0.3 ng/µl in the control group (p=0.08).

**Figure 1:**
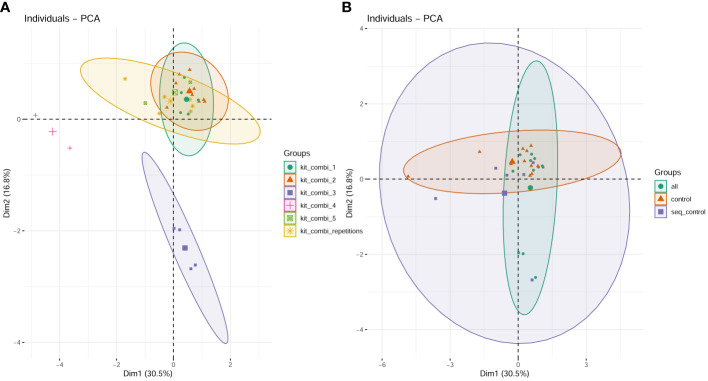
PCA plot visualizing the clustering of datasets based on the six investigated batches **(A)** compared to the distribution in the PCA plot based on the different groups (patients, controls and synthetic run controls) **(B)**.

**Figure 2:**
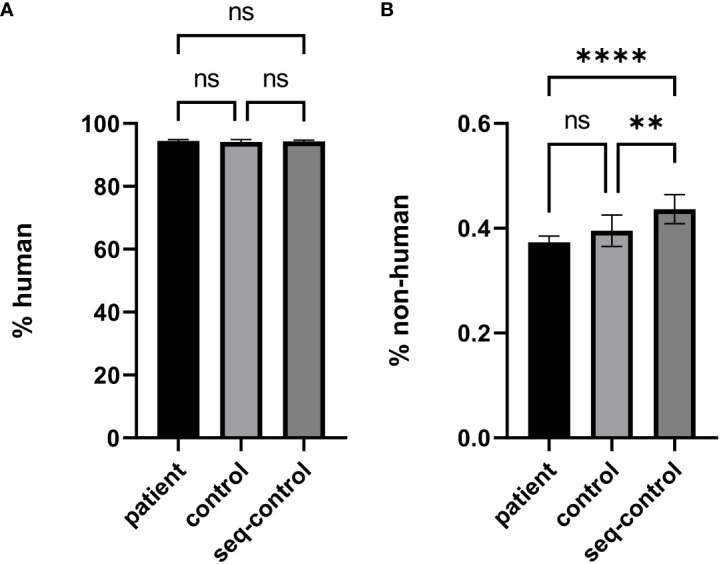
Comparison of mapped read proportions between the cohort of patients with newly diagnosed acute lymphatic leukemia (patient), the cohort of healthy donors and patients with non-oncologic diseases (control), and synthetic quality controls for the wetlab and sequencing process (seq-control) for human read fraction **(A)** and non-human read fraction **(B)**. **: p≤0.01, ****: p≤0.0001, ns: non significant.

The cfDNA samples were processed in 6 different batches with different reagent lots due to logistical requirements. The batches separate into different clusters (**Figure 1A**) in contrast to the plot showing the different groups (patients, controls, synthetic sequencing run controls, **Figure 1B**) where no separation based on any of these groups was possible. In the read mapping statistics regarding the proportion of human reads versus non-human reads (**Table 2**), no significant differences between the groups were discernible (**Figure 2A**), with the exception of the synthetic control that shows a statistically significant increase in the non-human proportion, due to the synthetic microbial sequences (**Figure 2B**). In blood and bone marrow samples of patients or controls, no significant amounts of viral DNA were identified. DNA fragments for Torque Teno virus were found in two patients in the raw data, but below LOD and reporting thresholds.

**Table 2:** Read mapping statistics of whole metagenome shotgun sequencing for all patient samples (n=23) in comparison to synthetic control samples.

Study ID	Patient	Group	Sample Type	total reads	total reads (%)	Unclassified reads (%)	Human reads (%)	Non-human reads (%)
A0006	3	patient	Blood	52,002,868	100.00%	5.64%	93.97%	0.39%
A0007	3	patient	BM	33,779,766	100.00%	6.32%	93.29%	0.39%
A0008	4	patient	Blood	32,735,894	100.00%	4.96%	94.67%	0.37%
A0009	4	patient	BM	32,188,415	100.00%	4.67%	94.98%	0.36%
A0014	7	patient	Blood	38,734,281	100.00%	5.11%	94.52%	0.38%
A0015	7	patient	BM	69,313,588	100.00%	5.21%	94.42%	0.38%
A0020	10	patient	Blood	40,929,261	100.00%	5.59%	94.03%	0.38%
A0021	10	patient	BM	34,978,231	100.00%	5.23%	94.41%	0.36%
A0022	11	patient	Blood	51,991,969	100.00%	5.05%	94.58%	0.37%
A0023	11	patient	BM	52,900,092	100.00%	5.13%	94.50%	0.37%
C0005	14	patient	Blood	33,459,164	100.00%	4.61%	95.01%	0.37%
C0006	14	patient	BM	37,141,333	100.00%	4.92%	94.72%	0.36%
*Mean (patients)*				*42,512,905*		*5.20%*	*94.43%*	*0.37%*
A0004	2	control	Blood	26,036,123	100.00%	5.21%	94.41%	0.39%
A0005	2	control	BM	26,543,185	100.00%	5.04%	94.61%	0.35%
A0010	5	control	Blood	68,049,522	100.00%	5.50%	94.08%	0.42%
A0011	5	control	BM	40,976,925	100.00%	7.14%	92.43%	0.43%
A0012	6	control	Blood	31,826,897	100.00%	4.47%	95.13%	0.40%
A0016	8	control	Blood	35,040,574	100.00%	5.06%	94.57%	0.37%
A0017	8	control	BM	32,807,936	100.00%	6.06%	93.51%	0.43%
A0018	8	control	Blood	29,234,374	100.00%	5.09%	94.55%	0.36%
A0019	8	control	BM	33,102,958	100.00%	4.98%	94.65%	0.36%
C0003	13	control	Blood	55,440,951	100.00%	5.64%	93.94%	0.41%
C0004	13	control	BM	46,684,511	100.00%	5.89%	93.69%	0.42%
*Mean (controls)*				*38,703,996*		*5.46%*	*94.14%*	*0.39%*
PK control sample	seq_control	seq_control		34,385,397	100.00%	5.15%	94.43%	0.42%
PK control sample	seq_control	seq_control		27,760,992	100.00%	5.45%	94.12%	0.43%
PK control sample	seq_control	seq_control		36,634,920	100.00%	5.40%	94.18%	0.42%
PK control sample	seq_control	seq_control		35,377,025	100.00%	5.78%	93.77%	0.45%
PK control sample	seq_control	seq_control		25,294,988	100.00%	5.26%	94.25%	0.49%
PK control sample	seq_control	seq_control		31,521,209	100.00%	4.49%	95.09%	0.42%
*Mean (synthetic controls)*				*31,829,089*		*5.26%*	*94.31%*	*0.44%*

BM, bone marrow.

## 4 Discussion

In this pilot trial, WMS of bone marrow samples in pediatric patients with treatment-naïve ALL revealed no specific infectious agents and no differences in the viral or bacterial metagenome when compared to peripheral blood of the same patients (internal validation) or in comparison to peripheral blood and BM of healthy children or children with non-oncologic diseases (external validation). As a limitation, only DNA viruses could have been identified, which could explain the opposing results of a higher viral infection load in ALL previously reported (Francis et al., 2014) where RNA has been amplified.

The absence of a discernible bacterial metagenome in the peripheral blood and bone marrow samples after implementation of rigid controls, could also be linked to the recent discussion about the hypothesized “human blood microbiome”, which has been postulated by several small trials using NGS techniques on peripheral blood samples (McLaughlin et al., 2002; Moriyama et al., 2008; Païssé et al., 2016), reviewed by Castillo et al. (2019) A frequent issue that arises from the combination of high-throughput sequencing and low-biomass samples (Castillo et al., 2019) is a contamination of the used DNA extraction kits with commensal bacteria (“kitome”) as well as cross-contamination between different samples in the laboratory (“splashome”) that might emulate a “microbiome”. This has been demonstrated by Olomu et al. (2020), who opposed the findings of a hypothetical placental microbiome (Bassols et al., 2016; Zheng et al., 2017) by adjusting for these confounders. Just recently, the idea of a “human blood microbiome” has been rendered unlikely by the findings of a population-based study including nearly 10,000 healthy subjects (Tan et al., 2023). However, the small cohort size of our pilot study as well as the fact that several patients received antibiotic treatment ahead of inclusion into this trial have to be acknowledged as important limitations in interpreting our data.

As a conclusion, further unbiased investigation of a potential, infectious-related etiology of pediatric ALL using untargeted DNA amplification methods should always implement rigid controls against potential confounders, and might consider the detection of viral DNA and RNA in larger sample sizes.

## Data availability statement

The data presented in the study are deposited in the NCBI BioProject repository, accession number PRJNA1123915.

## Ethics statement

The studies involving humans were approved by Ethical committee of Medical Faculty, University Tuebingen, approval number 561/2020BO1, gathered 11/2020. The studies were conducted in accordance with the local legislation and institutional requirements. Written informed consent for participation in this study was provided by the participants’ legal guardians/next of kin.

## Author contributions

ATH: Writing – review & editing, Writing – original draft, Validation, Investigation, Formal analysis, Data curation, Conceptualization. SG: Writing – review & editing, Writing – original draft, Validation, Software, Methodology, Investigation, Funding acquisition, Formal analysis, Data curation, Conceptualization. CS: Writing – review & editing, Writing – original draft, Validation, Methodology, Conceptualization. MD: Writing – review & editing, Data curation. MQ: Writing – review & editing, Data curation. RH: Writing – review & editing, Supervision, Project administration, Conceptualization. ME: Writing – review & editing, Supervision, Project administration, Data curation, Conceptualization.
