# Supplementary material for: No evidence on infectious DNA-based agents in pediatric acute lymphoblastic leukemia using whole metagenome shotgun sequencing
Source: Front Cell Infect Microbiol. 2024 Jun 21;14:1355787. doi: 10.3389/fcimb.2024.1355787 (PMC11224432; doi:10.3389/fcimb.2024.1355787)
Supplement: Supplementary file 1 [file DataSheet_1.docx]

Supplementary Material

No evidence on infectious DNA-based agents in pediatric acute lymphoblastic leukemia using whole metagenome shotgun sequencing

**Amadeus T. Heinz, MD^1,2^, Silke Grumaz, PhD^3^, Christoph Slavetinsky, MD, PhD^4^, Michaela Döring, MD^1^, Manon Queudeville^5^, MD, Rupert Handgretinger, MD^6^ and Martin Ebinger, MD*^1^**

*** Correspondence:** Prof. Martin Ebinger, MD

University Children’s Hospital of Tuebingen

Hoppe-Seyler-Str. 1, 72076 Tuebingen, Germany

Phone: +49 7071 29 83781

Mail: martin.ebinger@med.uni-tuebingen.de

**Supplementary Material 1**: Further information on validation of the DISQVR platform

Limit of detection (LOD) for DISQVER were determined within the scope of extensive analytical validations on the one hand for the bioinformatics pipeline and on the other hand for the total workflow, including wetlab. Validation of bioinformatics, dry-lab, pipelines have been done using in-silico as well as sequenced FASTQ data. In-silico data was prepared by randomly subsampling reads from different microbes representing bacteria, fungi, dsDNA viruses and parasites against different amounts of human DNA. The LOD has been determined to be independent of human DNA at 50 reads with a confidence of >= 95%. For the wetlab part, on 350 contrived samples, consisting of synthetic plasma matrix, human cfDNA in low-, medium and high input and pathogenic spikes of 3 different pathogens (bacterium, virus, fungus) a sensitivity of 99.99% with LOD of 41 pathogen molecules per microliter plasma was calculated (for medium human cfDNA background). Specificity was 99.99%. Repeatability with coefficient of variation < 50%, reproducibility with coefficient of variation < 35% and accuracy with coefficient of variation ≤ 40% were 100% each.

The sequencing run controls were prepared by using synthetic plasma matrix containing fragmented, human DNA from human cell lines and spiking these with known quantities of synthethic oligonucleotides from three different pathogens (bacterium, virus, fungus.)
